# Spatial Cognition of the Visually Impaired: A Case Study in a Familiar Environment

**DOI:** 10.3390/ijerph20031753

**Published:** 2023-01-18

**Authors:** Xinyi Zou, Ying Zhou

**Affiliations:** School of Architecture, Southeast University, No. 2 Sipailou, Nanjing 210096, China

**Keywords:** visually impaired, familiar environment, subjective cognition, physiological monitoring signal data, regular sequence of physical environment, spatial comfort

## Abstract

Objectives: This paper aims to explore the factors influencing the spatial cognition of the visually impaired in familiar environments. Background: Massage hospitals are some of the few places that can provide work for the visually impaired in China. Studying the spatial cognition of the visually impaired in a massage hospital could be instructive for the design of working environments for the visually impaired and other workplaces in the future. Methods: First, the subjective spatial cognition of the visually impaired was evaluated by object layout tasks for describing the spatial relationships among object parts. Second, physiological monitoring signal data, including the electrodermal activity, heart rate variability, and electroencephalography, were collected while the visually impaired doctors walked along prescribed routes based on the feature analysis of the physical environment in the hospital, and then their physiological monitoring signal data for each route were compared. The visual factors, physical environmental factors, and human–environment interactive factors that significantly impact the spatial cognition of visually impaired people were discussed. Conclusions: (1) visual acuity affects the spatial cognition of the visually impaired in familiar environments; (2) the spatial cognition of the visually impaired can be promoted by a longer staying time and the more regular sequence of a physical environment; (3) the spatial comfort of the visually impaired can be improved by increasing the amount of greenery; and (4) the visual comfort of the visually impaired can be reduced by rich interior colors and contrasting lattice floor tiles.

## 1. Introduction

According to the World Health Organization’s 2010 survey and estimate of the world population with a visual impairment, the number of those who are visually impaired is about 285 million worldwide, accounting for about 4.24% of the world’s total population, while 39 million people are blind and 246 million people are categorized as having low vision (LV) [1]. With the improvement in social inclusion for disabled groups, more and more visually impaired people are able to leave their homes more frequently and participate in social activities, finding their sense of self-worth in social life [2]. Research on friendly environments for the visually impaired could facilitate their faster integration into society. Exploring the relationship between spatial cognition and physical environmental factors has long been an important issue in the field of environmental behavior studies, since it may lead to enlightening design concepts [3]. Therefore, it is important to study the physical environmental factors influencing the spatial cognition of the visually impaired. Subjective investigations and objective measurements are often used in the field of environmental behavior studies [4,5].

Subjective investigations of the spatial cognition of the visually impaired can be traced back to 1951, when Philip Worchel designed an experiment to explore the differences in orientation awareness between visually impaired and sighted individuals [6]. After that, spatial orientation discrimination and the distance estimation abilities of the visually impaired have been studied several times, particularly with regard to the extent to which factors of gender [7], age [8,9], etiology [10], and congenital blindness [11,12] contribute to differences in the spatial cognition of the visually impaired. The spatial cognition of the visually impaired in different environments has also been discussed. J. F. Herman et al. (1983) designed a maze experiment to study the spatial cognitive ability of the visually impaired [13], and R. W. Byrne et al. (1983) conducted an experimental study of the spatial cognition of the visually impaired in a street environment [14]. In addition, an important role of tactile and auditory cues are proved on the environmental perception of the visually impaired [15,16]. The visually impaired cannot have the same visual experience as sighted people, even if they have been implanted with the Argus II retinal prosthesis, but they have learnt to rely on non-visual senses [17]. Many studies have attempted to help the cognitive space of the visually impaired through non-visual sensory tools. Non-visual multisensory integration could help the visually impaired to navigate their surroundings [18]. Similarly, an audiotactile map could help blind individuals become familiar with an area [19]. However, the understanding of space in these studies is limited, while studies on the cognition of an overall special structure have been greatly promoted due to cognitive maps [20,21]. Barbara Landau (1991) found that visual cues were not necessary for blind children to represent and transform spatial objects [22]. Cognitive map experiments have suggested that the visually impaired have a better understanding of small-scale spaces [23]. Furthermore, the visually impaired are regarded as having less ability to reorient themselves in spatial scaling, based on a study of the reversal errors of visually impaired people by Magdalena Szubielska (2019) [24]. Nevertheless, the issue of which kind of space (e.g., an open outdoor space, an enclosed indoor space, or a combination of indoor and outdoor spaces; a straight corridor or a corridor with a lot of turns) is most user-friendly for the visually impaired has not been discussed. Therefore, physical environmental factors that have an impact on the spatial cognition of the visually impaired need to be further studied through experiments.

In terms of the objective data on the spatial cognition of the visually impaired, indicators such as the correctness of spatial wayfinding [21,25,26], the ability to find the optimal route [6,27], the time and number of pauses in the routes [28], the task completion rate [28,29], and the time spent in wayfinding [29,30] have received attention in the literature. Romedi Passini et al. (1990) argued that people could form the structure of a maze in their brains despite the absence of vision, while age and education affected their wayfinding ability [31]. Subsequent studies have made significant advances with the development of anthropogenic ergonomics, finding that the interaction process between the visually impaired and the environment can be analyzed by recording small physiological changes that are not easily detectable. First, the eye tracking and attention points of LV individuals were recorded during a wayfinding experiment [32]. Second, EEG patterns were measured when the visually impaired touched cognitive maps [33]. Visually impaired and sighted people were asked to imagine that they were walking around a neighborhood near their home; meanwhile, their EEG patterns were monitored. The results suggest that the EEG alpha channel is more active in visually impaired people than in sighted people [34]. Similar studies have been conducted in the medical field in attempts to determine the reasons for behavioral differences between visually impaired and sighted individuals. Madeleine Fortin et al. (2008) found that the visually impaired had superior navigational skills when learning new paths in a maze setting, and a larger volume of the hippocampus of the visually impaired than sighted controls by magnetic resonance imaging (MRI) [23]. Additionally, Ron Kupersa et al. (2010) found that visually impaired individuals have the same cortical network as sighted individuals by functional magnetic resonance imaging (fMRI) [35]. The hippocampus and visual cortex of visually impaired individuals are activated during wayfinding, which was discovered by monitored blood oxygenation level-dependent (BOLD) fMRI [36].

The ways in which the visually impaired construct cognitive environments and the cues that influence their walking performance are generally considered to be of great importance to this group in previous studies. Previous research on the spatial cognition of the visually impaired is often conducted in unfamiliar environments, such as an unfamiliar building [2], eye clinic [32], and maze [31]. In these research areas, different visual acuity like low vision (LV) [27,32], light-shadow perception (LSP) [23,24], and no perception with light and shadow (NPLS) [24,34] was mentioned. However, these studies did not include visual acuity as an explanatory variable and did not explore the relationship between visual acuity and spatial cognition. It is proved that participants with LV cognized their environment by spatial characteristics, such as distinct floor edges and a high contrast, after a 30 min adaptation in the eye clinic [32], which helps to clarify that the length of stay in an environment could affect the spatial cognition of the visually impaired. In a maze, the visually impaired need to spend more time recognizing their environment than sighted people [31]. Compared to the usual environment, the maze is rich in variations of paths, but there is a real lack of variation in the environmental characteristic. However, familiar environments are rarely studied. Familiar environments such as the work environment, living environment, and learning environment are the places where visually impaired people spend most of their time, therefore familiar places are also worth studying. Massage hospitals are some of the few places that can provide work for the visually impaired in China, but they have not been studied so far. We proposed the following hypotheses: (1) the different visual condition of the visually impaired means they have a different spatial cognition in a familiar environment; (2) a different length of stay in a familiar environment results in the differing spatial cognition of the visually impaired, since interns work several months and doctors work several years; and (3) some physical environmental characteristics could help the visually impaired to recognize their environment and could interfere with their spatial cognition.

In summary, an experiment was designed to be conducted in the familiar work environment of visually impaired doctors, taking an integrated approach using both subjective investigations and objective measurements of spatial cognition. The subjective investigation of spatial cognition aimed at examining the understanding of the visually impaired regarding their surrounding space through object layout tasks and a verbal description. The objective measurement of spatial cognition aimed at evaluating the physiological performance of the visually impaired by collecting objective physiological signals, such as electrodermal activity (EDA), heart rate variability (HRV), and electroencephalography (EEG), while participants walked on designated routes, based on an analysis of the hospital building space, after which their physiological monitoring signal data were compared for each route. The application value of the research is to provide a new theoretical approach to the design orientation of architectural spaces for visually impaired users, thus informing the construction of massage hospitals and other workplaces for these users in the future.

## 2. Materials and Methods

### 2.1. Overview

**Figure 1:**
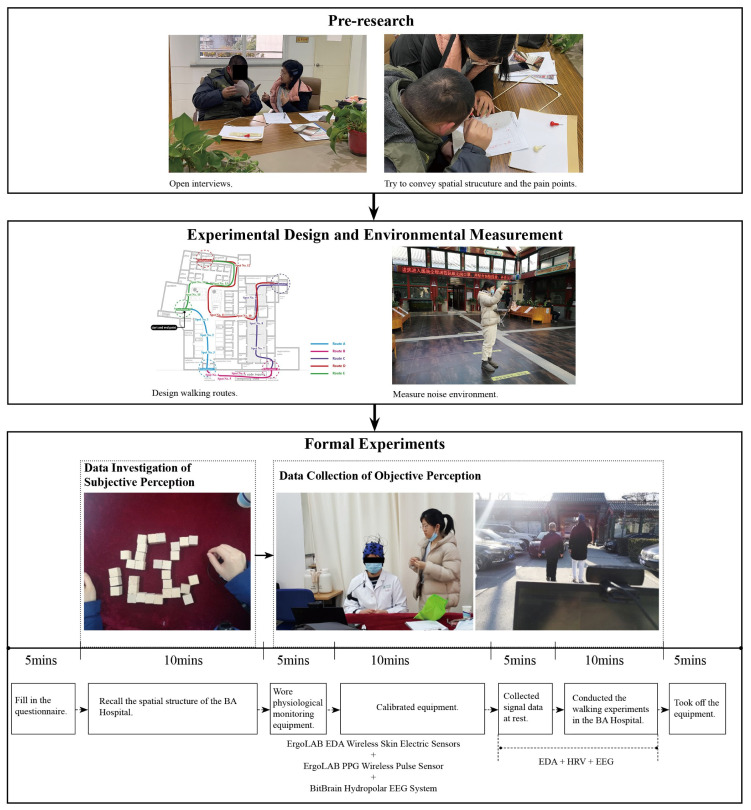
Flow chart of the overall research.

Before the formal experiment, we conducted open-ended interviews with 18 visually impaired doctors to ensure the feasibility and accuracy of the experiment. Then, the experimental walking routes were designed. We also measured the noise environment of the BA Hospital considering that pedestrians and the noise inside may have an impact on the experiment. The physical environmental measurement of the BA Hospital was conducted on 7 January 2022. The formal experiment was carried out from 10 to 13 January 2022. Oral communication was used to fill out the questionnaire, which solicited demographic and visual acuity information from the visually impaired. The formal experiment was divided into two parts: the subjective investigation of spatial cognition and the objective collection of spatial cognition. Each visually impaired doctor agreed to participate in a field trial of approximately 1 h in length. A flow chart of the overall research is shown in Figure 1.

### 2.2. Case Study

**Figure 2:**
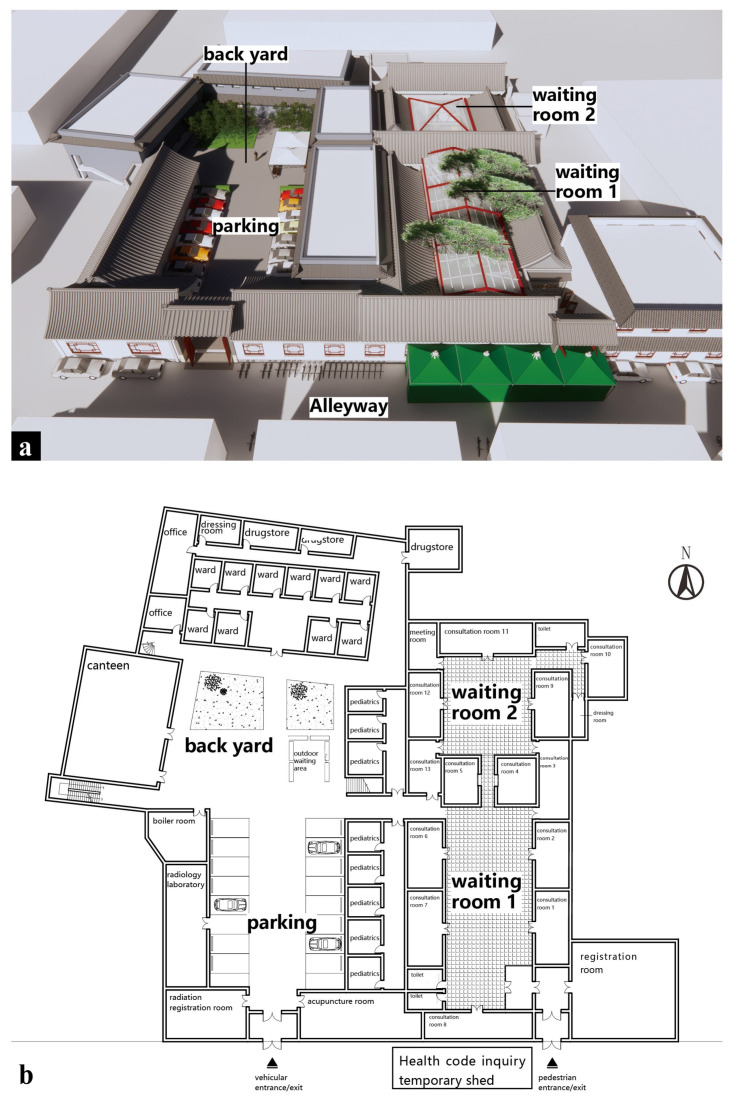
(**a**) Full view of the BA Hospital, (**b**) the BA Hospital floor plan.

The BA Hospital in the Xicheng District of Beijing, a massage hospital for the blind, is one of the main work environments for the visually impaired in China. Additionally, the BA Hospital is the birthplace of modern medical massage in China, giving it a certain scale and authority. Covering an area of 4000 square meters, the BA Hospital is constructed in a traditional Beijing courtyard style, with red walls, green trees, lattice floor tiles, old-fashioned crystal lights, and gorgeous murals intertwined together. Moreover, the combination of indoor and outdoor spaces leads to the diversity of the spatial sequence. A rendering of the BA Hospital is shown in Figure 2a, and the floor plan is shown in Figure 2b. Such a traditional architectural style is highly ornamental and interesting for sighted people, but whether it is friendly to the visually impaired remains to be studied.

### 2.3. Pre-Research

We conducted pre-research in October 2021. The transcripts of the open-ended interviews with 18 visually impaired doctors are shown in Table 1.

Routes for the walking tasks was developed based on the pain points of the BA Hospital space mentioned by the visually impaired doctors.

### 2.4. Preparation for Formal Experiment

#### 2.4.1. Design of Walking Routes

The design of the walking routes was based on the following principles. First, the routes were designed to pass through the problematic spaces mentioned by the visually impaired doctors during the pre-study and to pass through as many public spaces of different physical environmental types as possible. Second, each route was guaranteed to be of a certain length and duration to ensure the validity of the experimental data. Third, the order of the routes varied from easy to difficult, including the number of turning points and turning angles.

**Figure 3:**
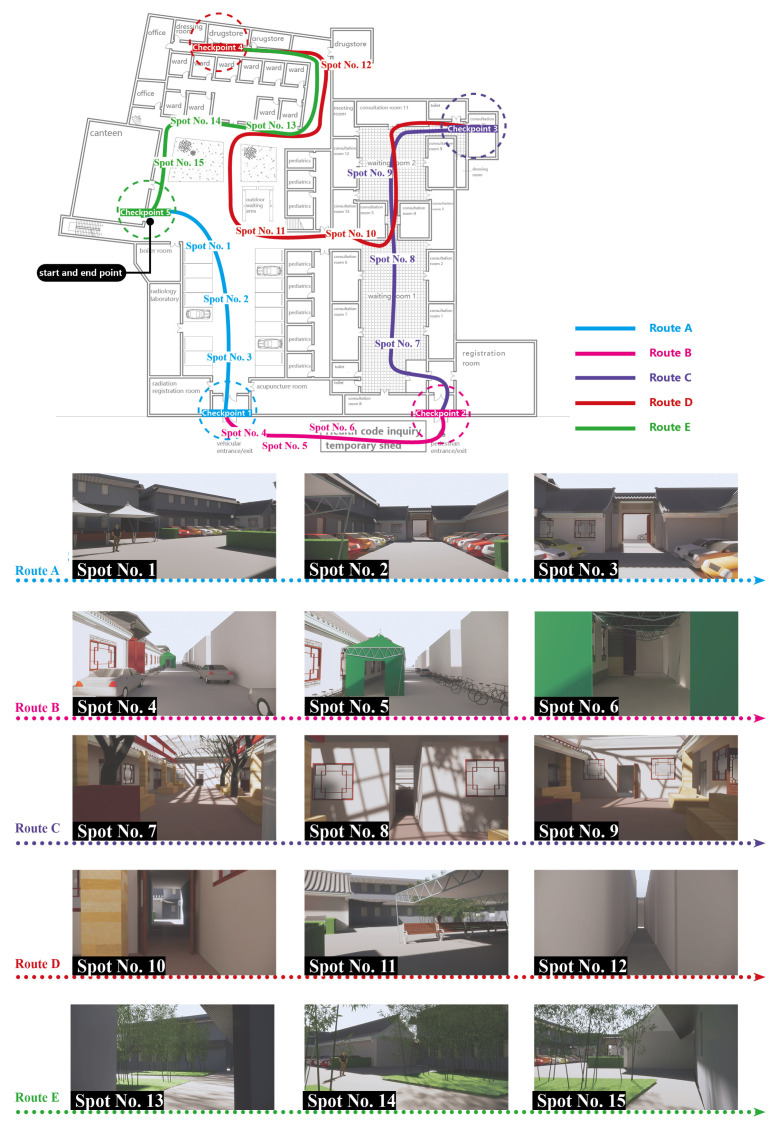
Routes of objective cognition measurements and the spot views.

The walking routes of the experiment are shown in Figure 3. The start point of the route was located at the canteen of the BA Hospital, and the end point returned to the start point. A total of five target points needed to be passed in the route, including the car entrance, the pedestrian entrance, consultation room 10, the pharmacy, and the canteen. The characteristics and the major issues of each route are shown in Table 2.

#### 2.4.2. Measurement of Noise Environment

The noise environment of the five routes was measured separately. Measurement points were set up every 4 m, starting from the beginning of each route, at a height of 1.6 m. A sound meter (BAPPU evo, ELK GmbH, Germany) was placed at the measurement points. The noise environment was measured three times per hour and was taken from 8:00 a.m. to 12:00 p.m. and from 14:00 p.m. to 17:00 p.m. This was the hospital’s business hours and the period in which the official experiment took place. 

### 2.5. Participants

There was a 3-month interval between the pre-research and the formal experiment. During the period, there was a job turnover among the blind doctors at the BA Hospital so that some intern doctors no longer worked at this hospital. We invited as many doctors as possible who participated in the pre-study to conduct the formal experiment, but we could not fully guarantee that they were the same group of people. Only 12 visually impaired doctors from the BA Hospital participated in the formal experiment. The demographic and visual acuity information from each participant is shown in Table 3. The study was conducted with the consent of all participants and was approved by the Medical Ethics Committee of the BA Hospital (No. 2022-02). Informed consent forms were signed with the participants before the experiment.

However, not all participants were able to complete the tasks. In object layout tasks, only 10 people were able to recover the spatial model of the BA Hospital. P1 and P11 could not complete the tasks. In walking tasks, only 7 participants completed all of the routes. P1 and P11 could not walk alone. P4, P5, and P8 did not complete all of the five routes.

### 2.6. Data Investigation of Subjective Cognition

Visually impaired doctors were provided with a number of cubic wooden blocks with a side length of 2.5 cm by the experimenters. They were asked to recall the spatial structure of the BA Hospital and to try to describe it while building a model of the hospital with the wooden blocks. At the same time, experimenters recorded the experiment process. After the experiment, the times of the spaces and corresponding descriptions mentioned by the visually impaired were recorded according to the experiment video. The descriptions of the BA Hospital by the visually impaired were summarized into five cognitive elements, the path, edge, district, node, and landmark, following Kevin Lynch’s method of classifying the elements of an urban space [37] (pp. 46–48).

### 2.7. Data Collection of Objective Cognition

The experimental apparatus was worn by the participant and then calibrated. The visually impaired doctors were asked to take a five-minute rest with their eyes open. After the rest, each participant was informed of five target locations to be reached and asked to walk at his or her usual pace. During the walking tasks, one experimenter followed the participant to ensure his or her safety, and another experimenter followed behind with a computer for the real-time transmission of the physiological experiment data and video recording of the experiment. Experimenters did not communicate with the participant verbally or physically in order to avoid disturbing physiological signals. After the walking tasks, the transmission of physiological monitoring data was stopped. The experimental equipment was removed from the participant. The whole experiment ended.

The ErgoLAB human–machine environment test cloud platform version 3.0 provided by Kingfar International Inc. was used in the walking tasks. Multimodal signal data, such as EDA, HRV, and EEG data, can be recorded by this platform simultaneously. The effectiveness of the ErgoLAB platform has been verified by researchers in related fields [38,39]. The contents of the multimodal signal indicators and their significance in this experiment are shown in Table 4.

### 2.8. Statistical Analysis

The physical measurement of the noise environmental data was performed on the BAPPU time software (ELK GmbH, Krefeld, Germany), which was for simultaneous long-term-recordings of health-relevant physical quantities. The average sound level of each route was calculated.

The physiological data, including the EDA, HRV, and EEG measured during the walking tasks, were preprocessed on the ErgoLAB platform. The fluctuation of the raw signal data is high due to the industrial frequency interference in the environment, so the noise data need to be filtered to remove the interference. Low-pass filtering, wavelet noise reduction, and Gaussian filtering preprocessing were performed on the EDA data. Low-wavelet noise reduction high/low-pass filtering, bandstop filtering, ectopic interval detection, and ectopic interval correction preprocessing were performed on the HRV data. Low-pass filter, high-pass filter, band-pass filter, and notch filter preprocessing were performed on the EEG data. All data were expressed as the mean ± standard deviation. All objective data collected by the physiological monitoring devices were statistically tested using SPSS 26.0.

## 3. Results

### 3.1. Noise Measurement

The average sound level of route A in each hour ranged from 53.4 dBA to 57.1 dBA (average 55.3 dBA). The average sound level of route B in each hour ranged from 53.5 dBA to 60.4 dBA (average 56.7 dBA). The average sound level of route C in each hour ranged from 57.7 dBA to 63.2 dBA (average 59.1 dBA). The average sound level of route D in each hour ranged from 50.5 dBA to 60.7 dBA (average 57.4 dBA). The average sound level of route E in each hour ranged from 51.3 dBA to 58.7 dBA (average 54.9 dBA). From the data results, we can see that the noise gap among the five routes is relatively small. Therefore, the effect of noise on the spatial cognition of the visually impaired was not focused on in this study.

### 3.2. Subjective Investigations of Spatial Cognition Based on the Object Layout Tasks

The results of the object layout tasks are shown in Table 5. Among the 12 visually impaired participants who participated in object layout tasks, 10 people were able to recover the spatial model of the BA Hospital, 1 person described the spatial structure of the BA Hospital, and 1 person did not have a spatial concept of the BA Hospital. The two participants who did not complete the spatial layout task were both interns with NPLS, which took up two-thirds of the total number of NPLS and one-fourth of the total number of interns. This is a difficult task for people with NPLS. Four visually impaired participants preferred to use “district” to describe the spatial structure of the BA Hospital. Apparently, district was the most commonly used element. Three visually impaired participants preferred to use “edge” to describe the spatial structure of the BA Hospital. Two visually impaired participants preferred to use “landmark” to describe the spatial structure of the BA Hospital. One visually impaired doctor preferred to use “path” to describe the spatial structure of the BA Hospital. There is a high variability in the type of cognitive spatial representation used by the visually impaired in the familiar environment.

The number of the visually impaired participants who mentioned space and corresponding descriptions are shown in Table 6. Most participants were able to describe the physical environmental characteristics of route C in the object layout tasks. They were able to describe the spatial sequence features from the large, rectangular waiting room to the narrow passage and then to the small, square waiting room, using multiple elements such as paths and districts to describe the spatial features of route C, summarizing the lines and surfaces comprehensively. It was shown that the visually impaired participants had a high level of spatial cognition while walking on route C. Five visually impaired participants described the physical environmental characteristics of route A as a vehicular entrance or parking lot, using node and district elements. It was shown that some of the visually impaired participants had a subjective cognition of the parking space in route A. About one-third of the participants described the physical environmental characteristics of route E as a backyard, yard, or flower bed, using landmark and district elements. Some of the visually impaired participants were able to perceive the presence of a green landscape. Route D was described by the participants using the path element. It was shown that the participants were less able to focus on this kind of traffic space.

### 3.3. Objective Measurements of Spatial Cognition Based on Walking Tasks

Among the five participants who did not complete all the walking tasks, P1 and P11, who were both interns with NPLS, could not walk alone; P5 only completed routes A, B, and C, and she was a doctor with NPLS; and P4 and P8 only completed routes A, B, C, and D; they were both interns with LSP. It was considered that 100% of LV participants were able to complete all the walking tasks, 50% of LSP participants were able to complete all the walking tasks, and no NPLS participant could complete all the walking tasks. Furthermore, 50% of interns were able to complete all the walking tasks and 75% of doctors were able to complete all the walking tasks. All interns stayed less than or equal to 6 months in the BA Hospital and all regular doctors stayed greater than or equal to 6 months in the BA Hospital. It was evident that the better the visual acuity and the length of stay in the work environment, the better the ability of the visually impaired to navigate a familiar environment. To ensure the data in each route were comparable, only the physiological signal data of the seven visually impaired participants who completed all the routes were analyzed.

#### 3.3.1. EDA

**Figure 4:**
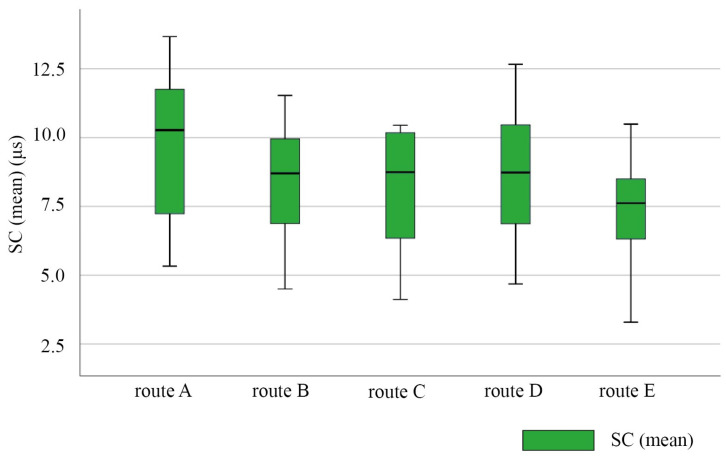
Box line diagram of SC average for the visually impaired on each route. N = 7.

The EDA signals could reflect the comfort level of the visually impaired. The skin conductance levels (SC) of visually impaired individuals while walking on each route are shown in Figure 4. The activity of the central nerve and sympathetic nerve of the visually impaired while walking on route A was the highest. Therefore, the level of sweat secretion activity of the sweat glands of the visually impaired was the strongest. This indicates that the visually impaired were the most stressed while walking on route A. The spatial environment of route A made the visually impaired feel the most uncomfortable. In contrast, the activity of the central nerve and sympathetic nerve of the visually impaired while walking on route E was the lowest. Therefore, the level of sweat secretion activity of the visually impaired was the weakest. This indicates that the visually impaired were the most relaxed while walking on route E. The spatial environment of route E made the visually impaired feel the most comfortable. The possible reason is that route A primarily traverses a parking space. For the visually impaired, places with traffic problems increase tension and stimulate their central nervous system. Further, route E had a higher percentage of greenery, leading to spatial comfort for the visually impaired. The skin conductance levels of the participants walking on routes B, C, and D had similar levels. These three routes were reported to induce a similar level of spatial comfort.

#### 3.3.2. HRV

**Figure 5:**
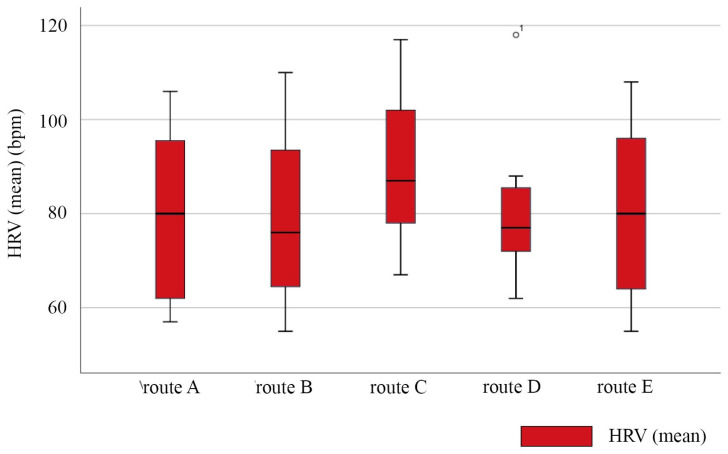
Box line diagram of HRV average for the visually impaired on each route. Circle and 1 in route D replaced there was an outlier in the statistics. N = 7.

The HRV signal could reflect the degree of emotional awakening of the visually impaired. The HRV levels of the participants while walking on each route are shown in Figure 5. The level of emotional awakening of the visually impaired was the highest on route C. This indicates that the level of emotional awakening of the visually impaired was the strongest. The possible reason was that the spatial environment of route C was colorful, energetic, and interesting. The presence of a crowd awakened the participants’ emotions. The HRV levels of the participants walking on routes A, B, D, and E were similar, showing that these four routes similarly affected the spatial interest of the visually impaired.

#### 3.3.3. EEG

**Figure 6:**
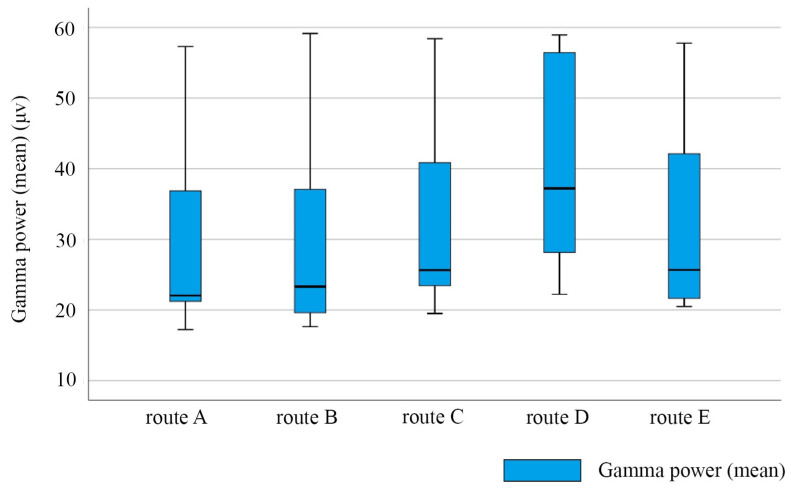
Box line diagram of Gamma power average for the visually impaired on each route. N = 7.

The Gamma power of the EEG signal could reflect the level of the brain activity of the visually impaired. The Gamma power of the visually impaired walking on each route is shown in Figure 6. The highest level of brain activity (total frequency of received EEG Gamma power) was observed in route D. Additionally, the individual differences in the brain activity of the visually impaired walking on route D were the strongest among the five routes. We found that the brains of the participants were most active while walking on route D. This indicated that the spatial environment of route D was the most complex and variable among the five routes. Furthermore, the level for the EEG Gamma power of the participants walking on route C was lower than those of the participants walking on route D. This indicated that route D was more identifiable than route C for the visually impaired. We found that route C had the most colorful interior decorative elements among all walking routes. Route D had a lot of turns and spatial changes, indicating that the visually impaired may have a stronger cognition of the spatial changes along the routes, rather than the decorative elements.

### 3.4. Comparative Studies of Subjective Investigations and Objective Measurements

The above results are purely subjective or objective, which may lead to them being less convincing. However, the comparative studies of subjective investigations and objective measurements can prove the results in two directions.

Judging from the completion status of the spatial layout tasks and walking tasks, the visually impaired have some commonality in their environmental cognitive abilities and walking abilities, as cognition is a prerequisite for action. All the participants who were able to complete the spatial layout tasks were able to walk alone in the BA hospital, but they were not necessarily able to complete all of the walking routes. In contrast, participants who were not able to complete the spatial layout tasks were not able to walk independently. From the aspect of the personal characteristics of the visually impaired who were unable to complete the task, it was shown that visual acuity and length of stay in the familiar environment had an effect on their spatial cognition and walking ability in a familiar environment.

Judging from the description of the subjective investigations and the statistic of objective measurements, a subjective cognitive description provides an interpretation of the objective measuring statistic. The subjective description and the mean values of the SC, HRV, and Gamma power of the participants walking on the five routes are shown in Table 7.

Comparing the mean SC values and mean HRV values of the visually impaired participants walking on routes D and E, we found that the mean HRV value of the participants walking on route D (81.71 ± 16.73 bpm) was similar to that of the participants walking on route E (80.43 ± 19.10 bpm). However, there was a large difference between the mean SC value of the participants walking on route D (8.68 ± 2.66 μS) and that of the participants walking on route E (7.29 ± 2.20 μS). The visually impaired were awakened to a similar extent on routes D and E, but the comfort levels of the visually impaired walking on route D were significantly different from those of the participants walking on route E. This indicated that the comfort levels of the visually impaired walking on route E were better than those of the participants walking on route D. From the aspect of the spatial structure of the walking routes, route D was similar to route E. However, the landscape greenery in route E accounted for more than half of the route distance, which was much higher than that in route D. In the spatial layout tasks, the visually impaired described route E as “backyard, flower beds”, reflecting their understanding of the remote location and green environment. However, their understanding of route D was simply “aisle”. Therefore, the result of the subjective description and the objective statistics indicated that the visually impaired gave more positive feedback regarding the landscape and a place with few people along route E.

Comparing the mean SC values and mean HRV values of the visually impaired individuals walking on routes A and B, we found that the mean SC value of the participants walking on route A (9.61 ± 2.92 μS) were higher than that of the participants walking on route B (8.34 ± 2.43 μS). Moreover, the mean HRV value of the participants walking on route A was higher (79.71 ± 19.17 bpm) than that of the participants walking on route B (79.57 ± 18.87 bpm). This indicated that the participants experienced a more emotional awakening on route A than they did on route B. In contrast, the participants were more uncomfortable on route A than on route B. This showed that even though the spatial interest of route A was higher than that of route B, the comfort of route B was higher than that of route A. In the spatial layout tasks, the visually impaired described route A as “vehicular entrance, parking lot”, reflecting their understanding of the traffic function of the space. From the aspect of the spatial structure of the routes, route A crossed an open and bright outdoor parking lot, while route B involved a dark and narrow temporary access running through a tent. For most of the visually impaired people with LV and LSP, route B was obviously depressing and dull. The reason why participants were still less comfortable with route A than route B was the traffic problem in the parking lot. For the visually impaired, spaces with cars make them feel nervous and uncomfortable. As a result, spatial safety is a prerequisite for spatial comfort and the most important need for the visually impaired.

Comparing the mean HRV value and the mean EEG Gamma power value of the participants walking on route C, we found that the mean HRV value of the visually impaired walking on route C (90.14 ± 16.65 bpm) was at the highest level among all of the routes. However, the EEG Gamma power value (33.17 ± 15.07 μV) of the visually impaired walking on route C was at the median level among the five routes. The subjective description of route C is the richest by the visually impaired, but the description is only based on the spatial characteristics and the sequence and does not provide a more in-depth description of the spatial details. However, they only had an average level of brain activity while walking on route C compared to the other routes. This indicated that although the visually impaired participants perceived the spatial environment of route C as the most interesting of the five routes, it did not effectively stimulate the brain activity. For people with LV, colorful spaces and stylized traditional architectural forms can increase their interest in the space, but overly complex decorations can disrupt their recognition of functional nodes in the space. Moreover, except for route C, the spatial interest of the other four routes increased with their spatial cognition. As a result, increasing the spatial interest of a public space helps the visually impaired to recognize spatial nodes, but spaces like route C that stimulate an excessive interest will hinder the visually impaired from recognizing a space.

## 4. Discussion

Most previous studies have separated the subjective spatial cognition research and the objective spatial cognition research of the visually impaired. However, a combination of these two aspects is necessary for a thorough and comprehensive understanding of how visually impaired people actually understand their environments. Some studies considered both aspects [23,29], but the differences in the spatial cognition of the visually impaired were explained only in terms of demographic and visual acuity information factors, without analyzing them from the perspective of physical environmental factors. We discuss the spatial cognition of the visually impaired from their visual, environmental, and human–environment interactive factors. 

### 4.1. Visual Factors Affecting Spatial Cognition of the Visually Impaired

The results of the object layout tasks and walking tasks showed that visual acuity could affect the spatial cognition of the visually impaired in familiar environments. Kazuhiko Mori et al. (2011) found that people gradually fail to recognize objects as their visual acuity decreases [2]. The conclusion of this paper is similar to Kazuhiko Mori’s conclusion.

### 4.2. Physical Environmental Factors Affecting Space Cognition of the Visually Impaired

#### 4.2.1. Spatial Characteristics

In the object layout tasks, route C had the highest mention rate and accuracy among all routes by the visually impaired participants. The spatial sequence and spatial morphology of route C was expressed by most of the visually impaired participants as “front-to-back” or “big-to-small.” The walking tasks showed that the visually impaired had a strong perception of spatial changes. Romedi Passini et al. concluded that the understanding of spatial geometric features by the visually impaired is not significantly different from that of sighted persons [20], which is similar to the findings of this paper. Didem Kan-Kilic et al. (2017, 2020) suggested that the visually impaired can perceive boundaries of space through their auditory senses, forming a sense of enclosure, so that they are able to subjectively identify the differences in the spatial dimensions and spatial shapes. A specific memory is produced when different forms of space are combined, forming a kind of special sequence [29,30]. Thus, people who are visually impaired can also comprehend the principle of spatial organization [41]. Visually impaired individuals with high spatial action abilities are quick to orient themselves by establishing interrelationships between spaces actively [25]. Furthermore, the conclusion that the visually impaired are able to have a positive feedback and awareness of the green landscape was found in both object layout tasks and walking tasks. The visually impaired can relax in a green landscape.

#### 4.2.2. Separation of Pedestrian and Vehicle

The spatial elements which can ensure the personal safety of visually impaired people, such as steps [42], color [43], and signage [44], have been the subjects of previous studies. The importance of pedestrian–vehicle segregation in enabling visually impaired people to walk freely, as a complement to the need for spatial safety, has been confirmed in this paper. The same findings were also found in interviews with stakeholders by Laura N. Cushley, who noted that vehicles were a potential hazard for visually impaired people [45].

#### 4.2.3. Decorative Elements

**Figure 7:**
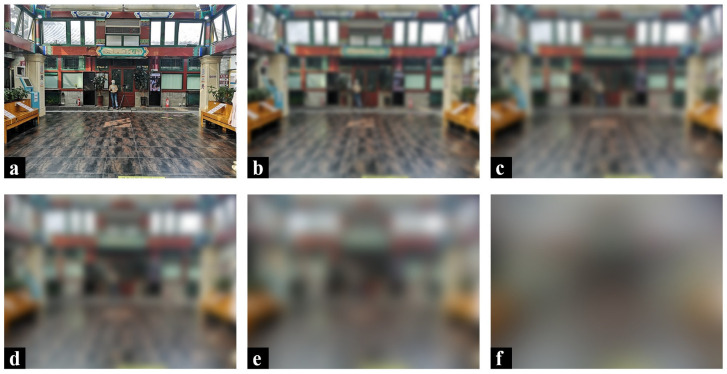
Simulation of the BA Hospital waiting room as seen by people with different levels of visual impairment: (**a**) unprocessed original image, (**b**) 30-pixel blurred image, (**c**) 50-pixel blurred image, (**d**) 70-pixel blurred image, (**e**) 120-pixel blurred image, (**f**) 280-pixel blurred image.

The excessive decoration of the waiting room in the BA Hospital does not promote the space recognition of the visually impaired. LV can be simulated by means of image processing, according to an experimental study by Kazuhiko Mori et al. (2011) [2]. Following their method to verify the findings of this paper, image processing by the blur (Gaussian radius) function of Adobe’s Photoshop software was used to reproduce the view of the waiting room in the BA Hospital, as seen by the visually impaired. The processing results in Figure 7 show that the overall vision of the waiting room was disturbed by the interior rich colors and the contrasting lattice floor tiles as the Gaussian blur radius increased. It is almost impossible to recognize elements in such a space, where the Gaussian blur radius is larger than 70 pixels (corresponding to a reproduced visual acuity of about 0.15), with the naked eye (Figure 7d). In addition, Yuji Matsuda et al. (2019) concluded that individuals with LV diverged in multiple directions while walking, and then their attention remained on the floor and floor’s edges. A certain width of boundary is friendly to people with LV [32]. Yuji Matsuda’s findings are complementary to the findings of this paper. In conclusion, rich colors and the contrasting lattice floor tiles in the room negatively affect the visual comfort of the visually impaired.

### 4.3. Human–Environment Interactive Factors

All those who did not complete the object layout tasks and the walking tasks were both interns. All of the interns had worked in the BA Hospital for less than 6 months. This shows that the length of stay in a familiar environment had an effect on their cognition and walking ability in a familiar environment. It is suggested that the length of stay in the work environment affects the visually impaired individuals’ spatial cognition and their ability to walk freely. No previous studies have been conducted in this area.

### 4.4. Future Research Directions

First, there is a paucity of research on the cognition of the visually impaired in familiar environments. Through this study, we have demonstrated a feasible method for subjective and objective research into the visually impaired in familiar environments. In the future, we will extend the method to a variety of work environments of the visually impaired. Second, we have demonstrated the extent to which visual acuity and the length of stay in a work environment affects the spatial cognition of the visually impaired in familiar environments. More factors of spatial cognition caused by the visually impaired themselves and the adaptation process of the visually impaired to familiar environments will be explored in the future. In addition, the environment in this study was a traditional Chinese courtyard, so modern spaces for the visually impaired need to be further explored. Finally, the visual comfort of the visually impaired has received less attention in previous studies. It will be considered in future studies.

### 4.5. Limitations

Despite the above findings, there are still limitations to the study. On the one hand, there is a small sample size due to scheduling conflicts that restricted the number of visually impaired doctors who could participate in the experiment. On the other hand, the visually impaired doctors working in this hospital generally have bachelor’s degrees or above and are 20–40 years old. The spatial cognitive ability of this young, educated visually impaired group may be more comprehensive than that of the general visually impaired group. The overall level of spatial cognition may be higher than that in other groups with different educational backgrounds or ages.

## 5. Conclusions

A combination of subjective investigations and objective collections of spatial cognition was used in this study, carried out in a hospital where visually impaired doctors had worked for a relatively long time. In addition to the functional use of the environments by the visually impaired, visual comfort was also considered. The results show that visual, environmental, and human–environment interactive factors affect the spatial cognition of the visually impaired.

## Figures and Tables

**Table 1:** The transcripts of the pre-study.

Number of Participants	Characteristics	Major Issues
18	Visual Acuity Low vision (LV): 9 people Light-shadow perception (LSP): 7 people No perception of light and shadow (NPLS): 2 people Length of stay in work environment Less than 0.5 year: 4 people 0.5 to 10 years: 5 people More than 10 years: 9 people	Visually impaired physicians have individual differences in their subjective perception of the hospital environment. Pain points: Visually impaired doctors reported some problems with the hospital: the decorative colors and floor tiles in the waiting room are confusing, the backyard of the hospital and the entrance to the hospital are dangerous because they mix people and vehicles, and the accessibility of the pharmacy is poor.

**Table 2:** Environmental characteristics of each walking route and the major issues.

	Characteristics	Major Issues
Route A	Outdoor space, mainly for parking, a necessary space for hospital entrances and exits.	Mixed traffic of people and vehicles is dangerous for the visually impaired.
Route B	The temporary tent set up in front of the hospital is where the hospital checks patients’ health codes during the COVID-19 pandemic.	The space is narrow and dimly lit, and the temporary functional space lacks interest.
Route C	The indoor space for patients waiting for consultation is in the traditional courtyard style with bright colors, which is highly ornamental and interesting.	Colorful interior decoration, which may cause dazzling.
Route D	It is an isolated area in the BA Hospital with a quiet environment.	Lots of changes and turns in this complex route.
Route E	It is an isolated area in the BA Hospital with a quieter environment and rich, green landscape.	The spot view of the route is dominated by gray exterior walls and greenery, which is not as interesting as the indoor space of the BA Hospital.

**Table 3:** Information of the participants.

Participants	Age (Years)	Gender	Occupation	Length of Stay in Work Environment	Visual Characteristics	Congenitally Blind	Pathogenesis
P1	24	Male	Intern	0.5	NPLS	YES	Unknown
P2	23	Male	Intern	0.5	LV	YES	Unknown
P3	22	Male	Intern	0.5	LV	YES	Unknown
P4	22	Male	Intern	0.5	LSP	NO	Retinitis pigmentosa
P5	37	Female	Doctor	3	NPLS	NO	Glaucoma
P6	27	Male	Doctor	2	LSP	YES	Retinitis pigmentosa
P7	26	Female	Doctor	0.5	LV	YES	Unknown
P8	22	Male	Intern	0.5	LSP	YES	Retinitis pigmentosa
P9	24	Male	Intern	0.5	LV	YES	Cataract
P10	21	Male	Intern	0.25	LV	NO	Retinitis pigmentosa
P11	21	Male	Intern	0.25	NPLS	YES	Retinitis pigmentosa
P12	28	Female	Doctor	3	LSP	NO	Unknown

N = 12.

**Table 4:** Content of multimodal signal index parameters and the significance in the experiment.

	Content	Significance
EDA Equipment name: ErgoLAB EDA Wireless Skin Electric Sensors Sampling rate: 64 Hz Accuracy: 0.01 μS Acquisition range: 0–30 μS Signal name: SC Average (μS)	Due to the steady-state character of the experimental procedure, without specific stimuli, a time-domain analysis was performed, using EDA time domain SC data to represent the skin conductance levels of visually impaired people over a period of travel [38,39,40]. In a person under duress, sweat secretion increases, resistance to the trace current through the sweat decreases, and SC increases. Conversely, in a relaxed person, the SC level decreases.	In this experiment, the tension or relaxation state of the participant in each route is illustrated. Thus, the comfort level of the environment is analyzed.
HRV Equipment name: ErgoLAB PPG Wireless Pulse Sensor Sampling rate: 64 Hz; Accuracy: 1% Acquisition range: 0–100% Signal name: HRV Average (bpm)	The mean values of HRV in the time domain were chosen to represent the level of heart rate variability of participants in each route [39,40]. HRV was closely related to emotional awakening. An increase in HRV was associated with an increase in emotion awakening and heart rate. A decrease in HRV was associated with a decrease in emotional awakening.	In this experiment, the degree of physical awakening of the participant in each route is illustrated. Thus, the extent to which the environment was interesting or monotonous is analyzed.
EEG Equipment name: BitBrain Hydropolar EEG System Sampling rate: 256 Hz Input range: ±100 mV; Input impedance: >50 GΩ Signal name: Gamma power Average (μV)	Gamma power, which is related to the events of this experiment, was selected for data analysis, representing the level of environmental perception of visually impaired people. Gamma power has an important role in the level of activity of the human brain and in higher activities such as transmission of information in the brain, integrated processing, and feedback [33,34]. It generally occurs when the brain performs cross-modal sensory processing tasks (e.g., synthesizing sound and light stimuli) or attempts to recall an object. It is susceptible to complex thinking operations.	In this experiment, the level of brain activity of the participants in each route is illustrated. Thus, the receptivity of participants to the environments is analyzed as easy or difficult to identify.

**Table 5:** Results of object layout tasks by the visually impaired.

Participant	Visual Characteristics	Occupation	Length of Stay in Work Environment	Result	Description Method
P1	NPLS	Intern	0.5 year	Uncompleted	/
P2	LV	Intern	0.5 year	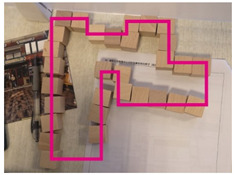	Edge
P3	LV	Intern	0.5 year	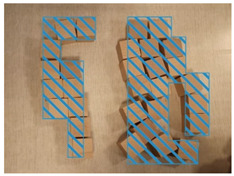	District
P4	LSP	Intern	0.5 year	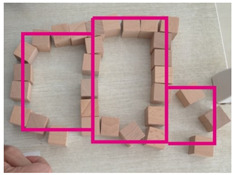	Edge
P5	NPLS	Doctor	3 years	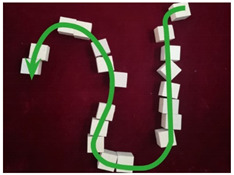	Path
P6	LSP	Doctor	2 years	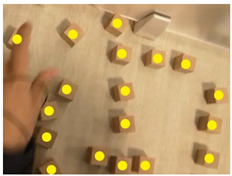	Landmark
P7	LV	Doctor	0.5 year	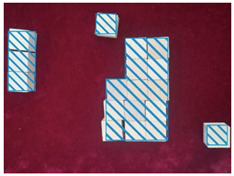	District
P8	LSP	Intern	0.5 year	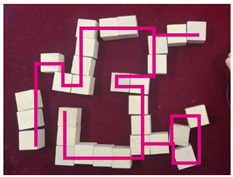	Edge
P9	LV	Intern	0.5 year	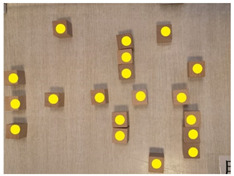	Landmark
P10	LV	Intern	0.25 year	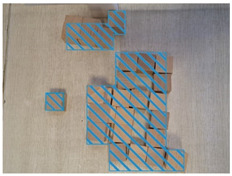	District
P11	NPLS	Intern	0.5 year	Uncompleted	/
P12	LSP	Doctor	3 years	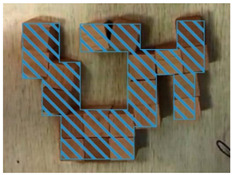	District

N = 10.

**Table 6:** The times of each walking route and their corresponding descriptive feature words.

	Number of the Visually Impaired Who Mentioned Space	Descriptive Feature Words	Elements
Route A	5 people	Vehicular entrance (LV, 0.5); Parking lot (LV, 0.5), (LSP, 0.5), (LSP, 3).	District, node
Route B	None	/	/
Route C	11 people	Large waiting room—aisle—small waiting room (NPLS, 3); Rectangle—narrow aisle—square (LV, 0.25); Big hall—aisle—small hall (LSP, 0.5); Front yard—aisle—backyard (LV, 0.5), (LSP, 2), (LSP, 0.5), (LSP, 3).	Path, district
Route D	2 people	Aisle (LV, 0.5), (LSP, 0.5)	Path
Route E	4 people	Backyard (LSP, 3), (NPLS, 3); Yard (LSP, 2); Flower beds (LV, 0.5).	District, landmark

N = 11.

**Table 7:** Mean values of SC, HRV, and Gamma power of the visually impaired on each route.

	Route A	Route B	Route C	Route D	Route E
Description	Vehicular entrance; Parking lot	/	Large waiting room—aisle—small waiting room; Rectangle—narrow aisle—square; Big hall—aisle—small hall; Front yard—aisle—backyard.	Aisle	Backyard; Yard; Flower beds
SC (μs)	9.61 ± 2.92	8.34 ± 2.43	8.05 ± 2.55	8.68 ± 2.66	7.29 ± 2.20
HRV (bpm)	79.71 ± 19.17	79.57 ± 18.87	90.14 ± 16.65	81.71 ± 16.73	80.43 ± 19.10
Gamma power (μV)	30.40 ± 14.87	30.49 ± 15.33	33.17 ± 15.07	41.07 ± 14.66	33.08 ± 15.18

Values are means ± standard deviation; N = 7.

## Data Availability

Not applicable.
